# Betacellulin induces Slug-mediated down-regulation of E-cadherin and cell migration in ovarian cancer cells

**DOI:** 10.18632/oncotarget.7591

**Published:** 2016-04-23

**Authors:** Jianfang Zhao, Christian Klausen, Xin Qiu, Jung-Chien Cheng, Hsun-Ming Chang, Peter C.K. Leung

**Affiliations:** ^1^ Department of Obstetrics and Gynaecology, Child & Family Research Institute, University of British Columbia, Vancouver, British Columbia V5Z 4H4, Canada

**Keywords:** betacellulin, ovarian cancer, E-cadherin, cell migration, Slug

## Abstract

Epithelial ovarian cancer is the leading cause of death among gynaecological cancers. Previous studies have demonstrated that epidermal growth factor receptor (EGFR) ligands can induce ovarian cancer cell invasion by down-regulating E-cadherin. Betacellulin is a unique member of the EGF family. It is overexpressed in a variety of cancers and is associated with reduced survival. However, the biological functions and clinical significance of betacellulin in ovarian cancer remain unknown. In the current study, we tested the hypothesis that betacellulin induces ovarian cancer cell migration by suppressing E-cadherin expression. Treatment of SKOV3 and OVCAR5 ovarian cancer cell lines with betacellulin down-regulated E-cadherin, but not N-cadherin. In addition, betacellulin treatment increased the expression of Snail and Slug, and these effects were completely blocked by pre-treatment with EGFR inhibitor AG1478. Interestingly, only knockdown of Slug reversed the down-regulation of E-cadherin by betacellulin. Betacellulin treatment induced the activation of both the MEK-ERK and PI3K-Akt signaling pathways, and it also significantly increased ovarian cancer cell migration. Importantly, the effects of betacellulin on E-cadherin, Slug and cell migration were attenuated by pre-treatment with either U0126 or LY294002. Our results suggest that betacellulin induces ovarian cancer migration and Slug-dependent E-cadherin down-regulation via EGFR-mediated MEK-ERK and PI3K-Akt signaling.

## INTRODUCTION

Ovarian cancer is the fifth most common cause of cancer-related death in women and the leading cause of death from gynaecological malignancies [1]. Ovarian cancer patients have a very low five-year survival rate (~45%) which is largely attributable to the high proportion of patients presenting with disseminated disease (~60%), for which the survival rate is only ~30% [1, 2]. Epidermal growth factor receptor (EGFR) is overexpressed in a variety of malignancies, including cancers of pancreas, breast, head and neck, lung and ovary [3]. In ovarian cancers, elevated expression of EGFR is correlated with poor prognosis [4–7]. EGFR belongs to the c-erbB receptor tyrosine kinase family, which includes 4 members: EGFR (ERBB1), ERBB2 (HER2), ERBB3 (HER3) and ERBB4 (HER4) [8]. Betacellulin (BTC) is an EGF-like growth factor that binds not only EGFR with high affinity, but also ERBB4 [9]. Overexpression of BTC has been found in many types of human cancers. [10–13]. In breast cancer, up-regulation of BTC is associated with reduced disease free survival [14]. In addition, BTC has been shown to act as an autocrine factor promoting the growth of pancreatic tumors[15]. BTC is an important regulator of ovarian follicle development, and has been shown to stimulate oocyte maturation and cumulus expansion [16]. BTC mRNA has been detected in ovarian tumors [17], however the functional role and clinical significance of BTC in ovarian cancer remains unknown.

E-cadherin, also known as cadherin 1 (CDH1), is a classical transmembrane cell-cell adhesion glycoprotein and a well-known tumor suppressor. E-cadherin plays an important role in maintaining normal epithelial cell polarity and structure [18, 19]. Down-regulation of E-cadherin and up-regulation of N-cadherin, often referred to as cadherin switching, is frequently associated with the process of epithelial-mesenchymal transition (EMT). EMTs involve the conversion of polarized, immotile epithelial cells to mesenchymal cells with a motile/invasive phenotype [20, 21]. In ovarian cancer, reduced total or cell surface E-cadherin expression is associated with poor overall or recurrence-free survival [22–24]. In addition, studies have shown that the expression of E-cadherin is negatively correlated with ovarian cancer cell invasiveness [25].

Our previous studies have shown that EGF induces ovarian cancer cell migration and invasion by down-regulating E-cadherin expression through a variety of signaling pathways [26–29]. Whereas BTC may function similar to EGF in many respects, its unique structure and receptor binding properties could result in unique mechanisms and functional roles. Our results show that BTC down-regulates E-cadherin expression and increases cell migration in an EGFR-dependent manner in two human ovarian cancer cell lines (SKOV3 and OVCAR5). Although BTC induces the expression of both Snail and Slug, two transcriptional repressors of E-cadherin, only Slug mediates its suppressive effects on E-cadherin expression. Moreover, our results show that both MEK-ERK1/2 and PI3K-Akt signaling pathways are involved in the effects of BTC on E-cadherin and cell migration.

## RESULTS

### BTC down-regulates E-cadherin, but not N-cadherin, via EGFR in ovarian cancer cells

To investigate the potential clinical relevance of BTC in ovarian cancer, we queried 489 ovarian cancers from The Cancer Genome Atlas (TCGA [30]) for up-regulation of BTC mRNA above the median. Kaplan-Meier analysis indicates that elevated levels of BTC mRNA are associated with reduced disease free survival (Log-rank *P*=0.0502, median 15.54 *vs.* 18.1 months; Supplementary Figure S1A) but not overall survival (Log-rank *P*=0.481; Supplementary Figure S1B). These results suggest that BTC could contribute to poor survival in ovarian cancer.

**Figure 1:**
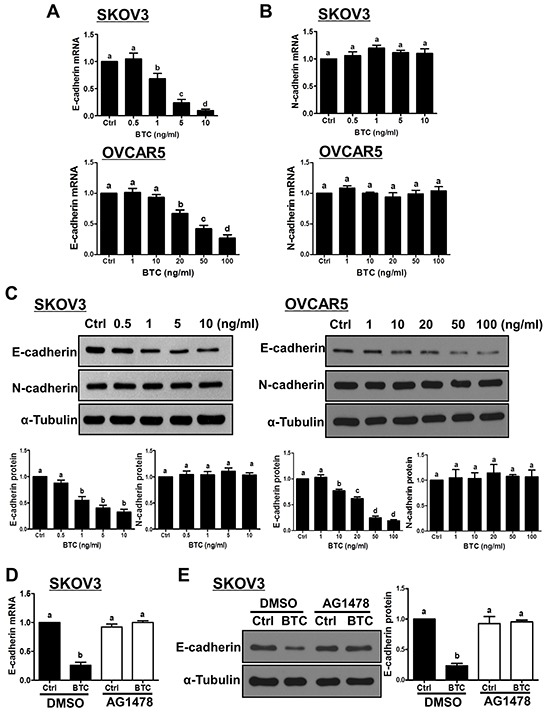
Betacellulin down-regulates E-cadherin, but not N-cadherin, via EGFR in ovarian cancer cells **A–C.** Cells were treated for 24 hours without (Ctrl) or with increasing concentrations of betacellulin (BTC: SKOV3, 0.5, 1, 5 or 10 ng/ml; OVCAR5, 1, 10, 20, 50 or 100 ng/ml), and E-cadherin (A) and N-cadherin (B) mRNA levels were examined by RT-qPCR. In addition, E-cadherin and N-cadherin protein levels (C) were examined by Western blot. **D–E.** SKOV3 cells were pre-treated for 1 hour with vehicle control (DMSO) or 10 μM AG1478 prior to treatment with or without 10 ng/ml BTC for 24 hours. E-cadherin mRNA (D) and protein (E) levels were examined by RT-qPCR and Western blot, respectively. Results are expressed as the mean ± SEM of at least three independent experiments and values without common letters are significantly different (*P*<0.05).

Next, we examined the effects of BTC on E-cadherin and N-cadherin expression in two ovarian cancer cell lines (SKOV3 and OVCAR5). As shown in Figure 1A, treatment for 24 hours with varying concentrations of BTC induced concentration-dependent reductions in E-cadherin mRNA levels in both cell lines, with SKOV3 cells displaying greater sensitivity. In contrast, treatment with BTC did not alter N-cadherin mRNA levels at any of the concentrations tested (Figure 1B). Western blot analysis confirmed the suppressive effects of BTC on E-cadherin, but not N-cadherin, protein levels in SKOV3 and OVCAR5 cells (Figure 1C). Next, we used the EGFR-specific inhibitor AG1478 to investigate the involvement of EGFR in BTC-induced E-cadherin down-regulation. As shown in Figure 1D and 1E, pre-treatment of SKOV3 cells with AG1478 completely blocked the down-regulation of E-cadherin mRNA and protein levels by BTC.

### BTC suppresses E-cadherin via Slug in ovarian cancer cells

**Figure 2:**
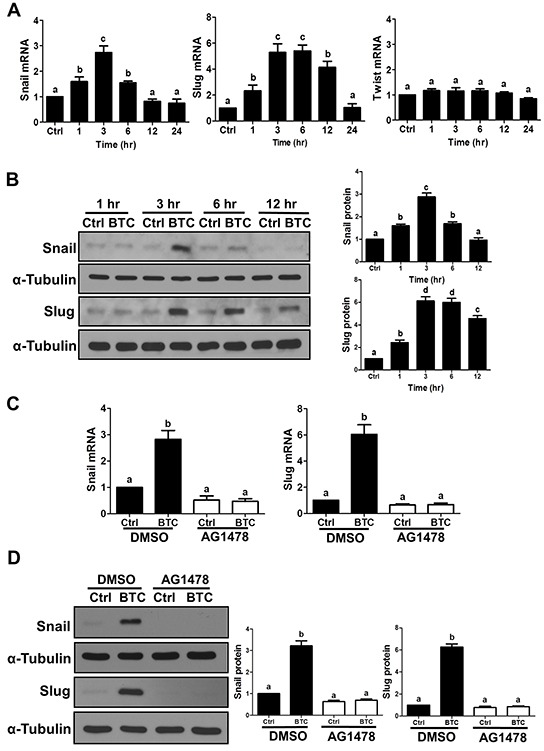
Betacellulin up-regulates Snail and Slug via EGFR in ovarian cancer cells **A–B.** SKOV3 cells were treated without (Ctrl) or with betacellulin (BTC: 10 ng/ml) for 1, 3, 6, 12 or 24 hours. A, Snail, Slug and Twist mRNA levels were examined by RT-qPCR. B, Snail and Slug protein levels were examined by Western blot. **C–D.** SKOV3 cells were pre-treated for 1 hour with vehicle control (DMSO) or 10 μM AG1478 prior to treatment with or without 10 ng/ml BTC for 3 hours. Snail and Slug mRNA (C) and protein (D) levels were examined by RT-qPCR and Western blot, respectively. Results are expressed as the mean ± SEM of at least three independent experiments and values without common letters are significantly different (*P*<0.05).

**Figure 3:**
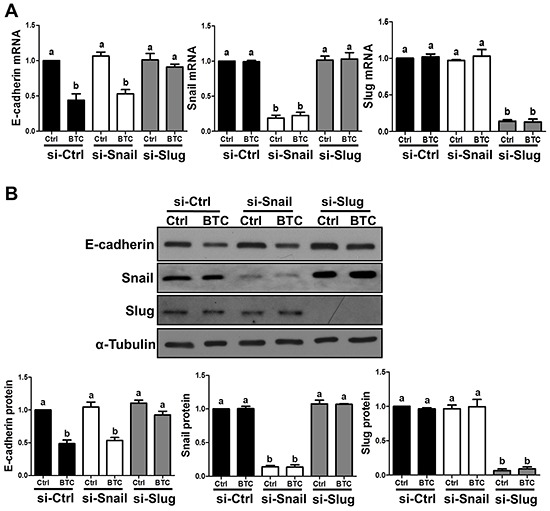
Betacellulin suppresses E-cadherin via Slug in ovarian cancer cells SKOV3 cells were transfected for 48 hours with 50 nM non-targeting control siRNA (si-Ctrl) or 50 nM siRNA targeting Snail (si-Snail) or Slug (si-Slug) prior to treatment for 24 hours without (Ctrl) or with 10 ng/ml betacellulin (BTC). E-cadherin, Snail and Slug mRNA **(A)** and protein **(B)** levels were examined by RT-qPCR and Western blot, respectively. Results are expressed as the mean ± SEM of at least three independent experiments and values without common letters are significantly different (*P*<0.05).

To investigate the involvement of Snail, Slug and/or Twist in BTC-induced E-cadherin down-regulation, we first examined the time-dependent effects of BTC on their mRNA and protein levels in SKOV3 cells. Whereas BTC treatment did not alter Twist mRNA levels (1, 3, 6, 12 or 24 hours; Figure 2A), it rapidly induced the mRNA and protein levels of both Snail and Slug, though the increases in Slug were more pronounced and sustained (Figure 2A and 2B). Consistent with our findings for E-cadherin, these BTC-induced increases in Snail and Slug mRNA and protein levels were abolished by pre-treatment with AG1478 (Figure 2C and 2D). To further confirm whether Snail or Slug mediates the suppression of E-cadherin by BTC, SKOV3 cells were transfected for 48 hours with Snail or Slug siRNA prior to treatment for 24 hours with BTC. RT-qPCR and Western blot analysis showed that whereas siRNA pre-treatment specifically down-regulated either Snail or Slug (Figure 3A and 3B), only knockdown of Slug blocked the suppressive effects of BTC on E-cadherin mRNA (Figure 3A) and protein (Figure 3B) levels.

### MEK-ERK and PI3K-Akt signaling contribute to the effects of BTC on E-cadherin and Slug

**Figure 4:**
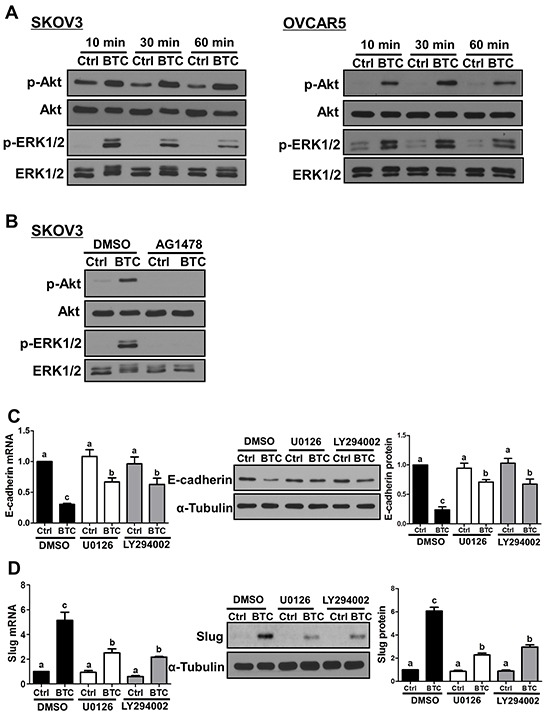
MEK-ERK and PI3K-Akt signaling contribute to the effects of betacellulin on E-cadherin and Slug **A.** Cells were treated without (Ctrl) or with betacellulin (BTC: SKOV3, 10 ng/ml; OVCAR5, 50 ng/ml) for 10, 30 or 60 minutes, and Western blot was used to measure the levels of phosphorylated Akt (p-Akt) and ERK1/2 (p-ERK1/2) in relation to their total levels (Akt and ERK1/2, respectively). **B.** SKOV3 cells were pre-treated for 1 hour with vehicle control (DMSO) or 10 μM AG1478 prior to treatment with or without 10 ng/ml BTC for 30 minutes. Western blot was used to measure the Akt and ERK1/2 phosphorylation/activation. **C–D.** SKOV3 cells were pre-treated for 1 hour with vehicle control (DMSO), 5 μM U0126 (MEK inhibitor) or 5 μM LY294002 (PI3K inhibitor) prior to treatment with or without 10 ng/ml BTC. E-cadherin (24 hours; C) and Slug (3 hours; D) mRNA and protein levels were examined by RT-qPCR and Western blot. Results are expressed as the mean ± SEM of at least three independent experiments and values without common letters are significantly different (*P*<0.05).

To investigate the involvement of MEK-ERK and PI3K-Akt signaling pathways in the effects of BTC on E-cadherin and Slug expression, we first used Western blot to examine their activation following treatment with BTC for 10, 30 or 60 minutes. As shown in Figure 4A, treatment with BTC increased the levels of phosphorylated Akt and ERK1/2 at all time-points in both SKOV3 and OVCAR5 cells. In addition, the effects of BTC on Akt and ERK1/2 phosphorylation were abolished by pre-treatment of SKOV3 cells with AG1478 (Figure 4B). Next, we used the MEK inhibitor U0126 and the PI3K inhibitor LY294002 to investigate the involvement of these two pathways in BTC-induced down-regulation of E-cadherin and up-regulation of Slug. Pre-treatment of SKOV3 cells with U0126 or LY294002 attenuated the suppressive effects of BTC on E-cadherin mRNA and protein levels (Figure 4C). Similarly, the up-regulation of Slug expression by BTC was attenuated by pre-treatment with U0126 or LY294002 (Figure 4D).

### BTC-induced ovarian cancer cell migration requires EGFR, MEK-ERK and PI3K-Akt signaling

**Figure 5:**
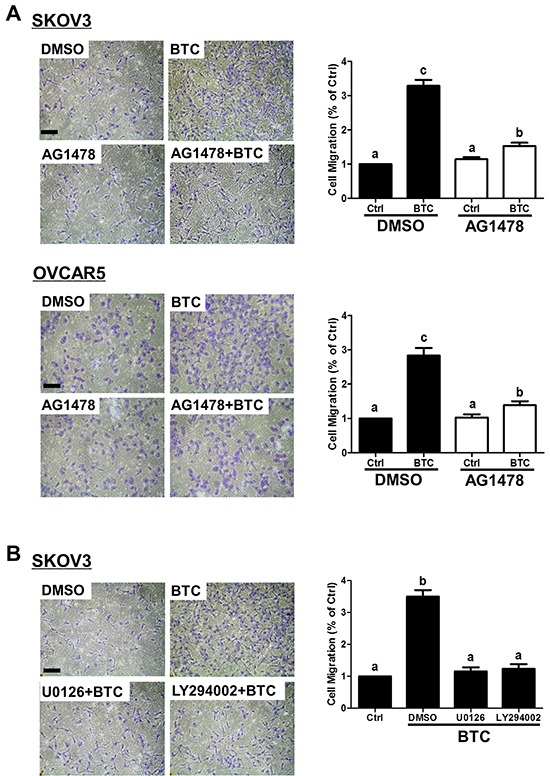
Betacellulin-induced ovarian cancer cell migration requires EGFR, MEK-ERK and PI3K-Akt signaling **A.** SKOV3 and OVCAR5 cells were pre-treated for 1 hour with vehicle control (DMSO) or 10 μM AG1478 prior to treatment without (Ctrl) or with betacellulin (BTC: 10 ng/ml for SKOV3, 50 ng/ml for OVCAR5), and cell migration was examined by transwell assay (12 hours). **B.** SKOV3 cells were pre-treated for 1 hour with or without 5 μM U0126 or 5 μM LY294002 prior to treatment with 10 ng/ml BTC, and cell migration was examined by transwell assay (12 hours). Results are expressed as the mean ± SEM of at least three independent experiments and values without common letters are significantly different (*P*<0.05).

We have previously shown that loss of E-cadherin contributes to EGF-induced ovarian cancer cell invasiveness [27, 31, 32]. To determine whether BTC induces similar pro-migratory effects, SKOV3 and OVCAR5 cells were treated for 12 hours with BTC and then subjected to transwell migration assays. As shown in Figure 5A, BTC treatment increased the migration of both SKOV3 and OVCAR5 cells, and this effect was almost completely abolished by pre-treatment AG1478. In addition, pre-treatment with U0126 or LY294002 reversed the stimulatory effects of BTC on SKOV3 cell migration (Figure 5B).

## DISCUSSION

The fact that most ovarian cancers are diagnosed at advanced stage with widespread peritoneal dissemination is the primary reason for their high mortality, and a persistent therapeutic challenge [1, 33]. EGF-like growth factors have been shown to enhance the invasiveness of ovarian cancer cells by suppressing the expression of E-cadherin [27, 32, 34]. Also a member of this family, BTC has been detected in various human cancers [10–13], where it has been shown to modulate cancer cell growth, invasion and resistance to targeted therapeutics [35–37]. To date, the potential role of BTC in ovarian cancer remains poorly defined. Previous studies by Tanaka *et al.* failed to show a significant difference in BTC mRNA between normal ovary and ovarian tumors [17], however most ovarian cancers are thought to arise from the fallopian tube epithelium or the ovarian surface epithelium [38, 39], and comparisons of BTC expression to these cell types have not been reported. Interestingly, however, their results did suggest a trend towards increased BTC expression in stage III-IV tumors [17]. These results are in agreement with our findings that BTC treatment promotes ovarian cancer cell motility, and that BTC is associated with reduced disease free survival. Together with previous studies, our results suggest that enhanced BTC signaling may contribute to ovarian cancer progression, though future studies are required to fully characterize its functional roles and molecular determinants.

BTC has been shown to induce head-and-neck squamous carcinoma cell invasion by up-regulating MMP9 [12, 36]. BTC has also been suggested to induce pancreatic islet migration by modulating RAC1 activity [40]. We now report, for the first time, that BTC induces ovarian cancer migration by down-regulating E-cadherin expression. Besides its putative roles in cancer cell migration/invasion, BTC has also been linked to other processes related to the hallmarks of cancer. For example, several groups have demonstrated the pro-proliferative effects of BTC in pancreatic cancer [15, 41, 42]. Moreover, BTC has been implicated in the development of an inflammatory microenvironment in lung cancer [43]. In addition, BTC has been shown to induce the proliferation and migration of vascular smooth muscle and umbilical vein endothelial cells, indicating a potential role for BTC in angiogenesis [44, 45]. Given that BTC could contribute to poor survival in ovarian cancer, future studies investigating the roles of BTC in ovarian cancer cell invasion, proliferation, apoptosis and angiogenesis would be of interest.

EGF-like growth factors elicit their effects by binding to and activating ERBB receptor homo- or heterodimers [46]. BTC has unique receptor binding properties compared to other well-studied EGF-like growth factors that bind exclusively to EGFR (e.g. EGF, transforming growth factor-α, amphiregulin). In particular, BTC can bind to either EGFR or ERBB4 and subsequently activate their respective homodimers or all the possible ERBB heterodimers [9]. Previous studies have used AG1478 and an EGFR-specific antagonistic antibody (ICR-62) to demonstrate the importance of EGFR in mediating BTC-induced cell migration and invasion [36, 45]. Similarly, we found that pre-treatment with AG1478 fully blocked BTC-induced E-cadherin down-regulation, Snail and Slug expression, and ERK1/2 and Akt activation. However, BTC-induced SKOV3 and OVCAR5 cell migration was only partially inhibited by AG1478, suggesting a potential role of ERBB4 in BTC-induced ovarian cancer migration. ERBB4 is the least investigated of all the ERBB family members in ovarian cancer, especially with regards to the effects of BTC. However, several groups have studied the expression and clinical importance of ERBB4 in ovarian tumors [47–50]. Interestingly, mounting evidence suggests that different isoforms of ERBB4 may correlate with different clinical outcomes in ovarian cancer patients [51, 52]. Thus, the roles of BTC and ERBB4 in ovarian cancer are likely complex, and warrant further investigation.

Loss of E-cadherin is a key event in epithelial-mesenchymal transition and is associated with poor overall or recurrence-free survival in ovarian cancer [22–24]. We have previously investigated the roles of several E-cadherin transcriptional repressors in mediating the effects of EGF-like growth factors on E-cadherin expression and invasion in ovarian cancer cells [27, 32, 34]. In particular, whereas both Snail and Slug are involved in EGF- and amphiregulin-induced E-cadherin down-regulation, Snail does not participate in the effects of transforming growth factor-α [34]. Interestingly, we show that, like transforming growth factor-α, BTC-induced E-cadherin down-regulation involves Slug, but not Snail. Thus, many of the EGFR-mediated functions of BTC are likely to be similar to other EGF-like growth factors, however BTC could induce a novel subset of effects via ERBB4. In addition to Snail and Slug, we have recently shown that hypoxia-inducible factor-1α, a key regulator of hypoxic responses [53], also mediates EGF-induced E-cadherin down-regulation and ovarian cancer cell invasion [29]. Interestingly, hypoxia-inducible factor-1α has been shown to participate in BTC-driven mesenchymal stem cell proliferation [54]. Future studies will be required to examine the effects of BTC on hypoxia-inducible factor-1α expression in ovarian cancer cells, and whether it may contribute to adaptation to hypoxia, proliferation and/or metastasis.

In summary, our study demonstrates that BTC signals through EGFR to up-regulate Snail and Slug in a MEK-ERK- and PI3K-Akt-dependent manner. Elevation of Slug, not Snail, is required for the down-regulation of E-cadherin expression which promotes ovarian cancer cell migration.

## MATERIALS AND METHODS

### Cell culture

SKOV3 and OVCAR5 human epithelial ovarian cancer cell lines were obtained from American Type Culture Collection. Cells were incubated in a 1:1 (vol/vol) mixture of M199/MCDB105 medium (Sigma-Aldrich) supplemented with 10% (vol/vol) fetal bovine serum (FBS; Hyclone Laboratories) and 1% (vol/vol) penicillin/streptomycin (Gibco). Cells were cultured at 37°C in a humidified atmosphere containing 5% CO_2_ and 95% air, and were serum starved for 24 hours prior to treatment.

### Antibodies and reagents

The monoclonal antibodies used in this study were: anti-human E-cadherin (36/E-cadherin, BD Biosciences), anti-human N-cadherin (32/N-cadherin, BD Biosciences), anti-porcine α-tubulin (B-5-1-2, Santa Cruz Biotechnology), anti-human Snail (L70G2, Cell Signaling Technology), and anti-human phospho-ERK1/2 (Thr202/Tyr204) (E10, Cell Signaling Technology), anti-human Slug (C19G7, Cell Signaling Technology). The polyclonal antibodies used were: anti-rat ERK1/2 (9102, Cell Signaling Technology), anti-human phospho-Akt (9271, Cell Signaling Technology), anti-mouse Akt (9272, Cell Signaling Technology). The horseradish peroxidase-conjugated goat anti-mouse IgG and goat anti-rabbit IgG were obtained from Bio-Rad Laboratories. *E. coli*-derived recombinant human betacellulin (Asp32-Tyr111) was obtained from R&D Systems. Human recombinant epidermal growth factor (E9644), AG1478 and LY294002 were obtained from Sigma-Aldrich. U0126 was obtained from Calbiochem.

### Small interfering RNA (siRNA) transfection

To knock down endogenous Snail or Slug, cells were plated at low density, allowed to recover for 24 hours, and then transfected with 50 nM ON-TARGET*plus*SMARTpool siRNA (Dharmacon) using Lipofectamine RNAiMAX (Invitrogen) according to the manufacturer's instructions. ON-TARGET*plus* non-targeting control pool siRNA (50 nM; Dharmacon) was used as a transfection control in all experiments.

### Reverse transcription-quantitative real-time PCR (RT-qPCR)

Total RNA was extracted using TRIzol Reagent (Invitrogen) according to the manufacturer's instructions. Reverse transcription was performed with 3 μg of RNA, random primers, and Moloney murine leukemia virus reverse transcriptase (Promega). SYBR Green RT-qPCR was performed on Applied Biosystems 7300 Real-Time PCR System equipped with 96-well optical reaction plates. Each 20 μl RT-qPCR reaction contained 1×SYBR Green PCR Master Mix (Applied Biosystems), 20 ng cDNA and 150 nM of each specific primer. The primers used were: E-cadherin (*CDH1*), 5′-ACA GCC CCG CCT TAT GAT T-3′ (sense) and 5′-TCG GAA CCG CTT CCT TCA-3′ (antisense); N-cadherin (*CDH2*), 5′-GGA CAG TTC CTG AGG GAT CA-3′ (sense) and 5′-GGA TTG CCT TCC ATG TCT GT-3′ (antisense); Snail (*SNAI1*), 5′-CCCCAATCGGAAGCCTAACT-3′ (sense) and 5′-GCTGGAAGGTAA ACT CTG GAT TAG A-3′ (antisense); Slug (*SNAI2*), 5′-TTC GGACCC ACA CAT TAC CT-3′ (sense) and 5′-GCAGTGAGGGCAAGA AAA AG-3′ (antisense); Twist (*TWIST1*), 5′-GGA GTC CGC AGT CTT ACG AG-3′ (sense) and 5′-TCT GGA GGA CCT GGT AGA GG-3′ (antisense); and glyceraldehyde-3-phosphate dehydrogenase (*GAPDH*), 5′-GAG TCA ACGGAT TTG GTC GT-3′ (sense) and 5′-GAC AAG CTT CCC GTTCTC AG-3′ (antisense). The amplification parameters were 50°C for 2 minutes, 95°C for 10 minutes, and 40 cycles of 95°C for 15 seconds and 60°C for 1 minute. At least three separate experiments were performed on different cultures and each sample was assayed in triplicate. A mean value was used for the determination of mRNA levels by the comparative Cq method (2^−ΔΔCq^) with GAPDH as the reference gene.

### Western blots

Cells were lysed in lysis buffer (Cell Signaling Technology) containing protease inhibitor cocktail (Sigma-Aldrich). Lysates were centrifuged at 20,000×*g* for 10 minutes at 4°C and supernatant protein concentrations were quantified using the DC Protein Assay (Bio-RadLaboratories) with BSA (A4503, Sigma-Aldrich) as the standard. Equal amounts of protein were separated by SDS-PAGE and transferred to polyvinylidene fluoride membranes. After blocking with Tris-buffered saline containing 5% non-fat dry milk for 1 hour, membranes were incubated overnight at 4°C with primary antibodies E-cadherin (1:3000), N-cadherin (1:3000), α-Tubulin (1:3000), phospho-ERK1/2 (1:3000), ERK1/2 (1:3000), phospho-Akt (1:3000), Akt (1:3000), Snail (1:1000) or Slug (1:1000), followed by incubation with the peroxidase-conjugated secondary antibody (1:5000). Immunoreactive bands were detected with enhanced chemiluminescent or SuperSignal West Femto substrate (Pierce). Membranes were stripped with stripping buffer (50 mM Tris-HCl pH 7.6, 10 mM β-mercaptoethanol and 1% SDS) at 50°C for 30 minutes and reprobed with anti-α-Tubulin, anti-ERK1/2 or anti-Akt as a loading control. Immunoreactive band intensities were quantified by densitometry, normalized to those of the relevant loading control, and the results are expressed as fold change relative to the respective control.

### Transwell migration assays

Migration assays were performed in Boyden chambers with minor modifications [55]. Transwell cell culture inserts (24-well, pore size 8 μm; BD Biosciences) were seeded with 1×10^5^ cells in 250 μL of medium with 0.1% FBS. Medium with 10% FBS (750 μl) was added to the lower chamber and served as a chemotactic agent. After 12 hours incubation, non-migrating cells were wiped from the upper side of the membrane and cells on the lower side were fixed in cold methanol (−20°C) and air dried. Cells were stained with Crystal Violet and counted using a light microscope (10× objective) equipped with a digital camera (QImaging) and Northern Eclipse 6.0 software. Five microscopic fields were counted per insert, triplicate inserts were used for each individual experiment, and each experiment was repeated at least three times.

### Statistical analysis

Results are presented as the mean ± SEM of at least three independent experiments. PRISM software (GraphPad Software Inc.) was used to perform one-way ANOVA followed by Tukey's multiple comparison test. Means were considered significantly different if *P* < 0.05 and are indicated by different letters.

## SUPPLEMENTARY FIGURE


