# Supplementary material for: Betacellulin induces Slug-mediated down-regulation of E-cadherin and cell migration in ovarian cancer cells
Source: Oncotarget. 2016 Apr 23;7(20):28881–90. doi: 10.18632/oncotarget.7591 (PMC5045363; doi:10.18632/oncotarget.7591)
Supplement: Supplementary file 1 [file oncotarget-07-28881-s001.pdf]

Betacellulin induces Slug-mediated down-regulation of E-cadherin and cell migration in ovarian cancer cells

SUPPLEMENTARY FIGURE

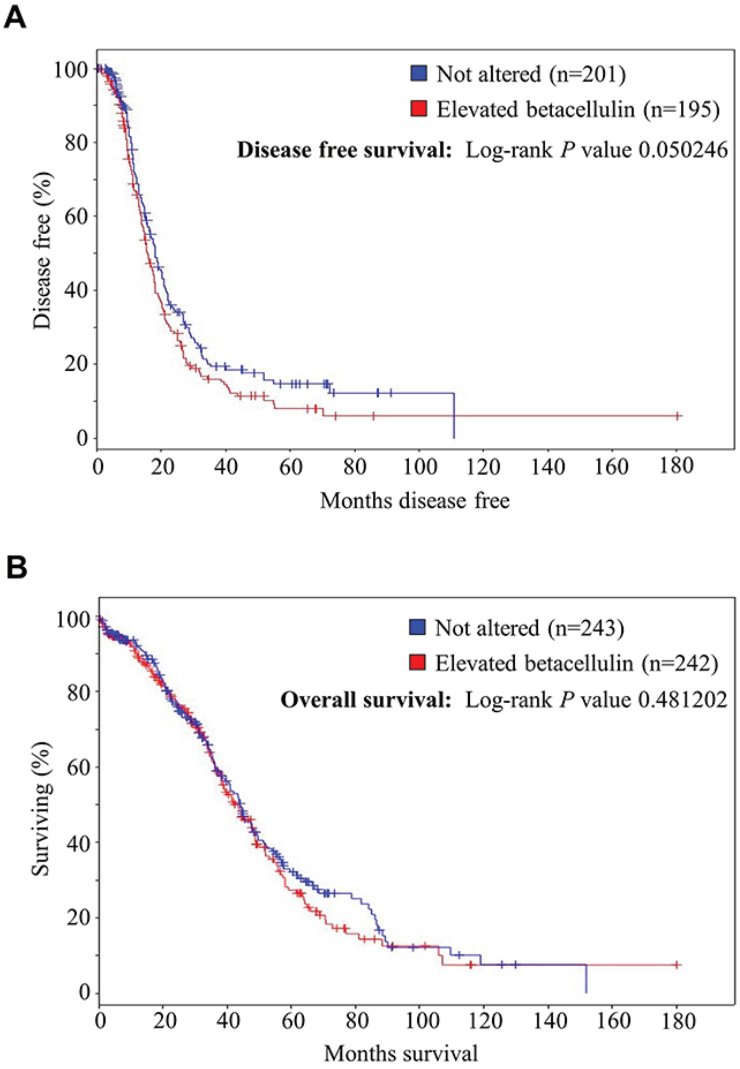

Supplementary Figure S1: Elevated betacellulin is associated with reduced disease free survival but not overall survival in ovarian cancers. The cBioPortal for Cancer Genomics was used to query ovarian carcinomas from The Cancer Genome Atlas (n=489) for up-regulation of betacellulin mRNA above the median. Disease free **A.** and overall **B.** survival differences between unaltered samples and those with elevated betacellulin are displayed as Kaplan-Meier survival curves with a *P* value from a Log-rank test.
